# Socioenvironmental factors associated with heat and cold-related mortality in Vadu HDSS, western India: a population-based case-crossover study

**DOI:** 10.1007/s00484-017-1363-8

**Published:** 2017-05-19

**Authors:** Vijendra Ingole, Sari Kovats, Barbara Schumann, Shakoor Hajat, Joacim Rocklöv, Sanjay Juvekar, Ben Armstrong

**Affiliations:** 1 Vadu Rural Health Program, KEM Hospital Research Centre, Pune, 411011 India; 2 Epidemiology and Global Health, Department of Public Health and Clinical Medicine, Umeå University, Umeå, Sweden; 3 Graduate School in Population Dynamics and Public Policy, Umeå University, Umeå, Sweden; 4 Centre for Demographic and Aging Research, Umeå University, Umeå, Sweden; 5 Department of Social and Environmental Health Research, London School of Hygiene and Tropical Medicine, London, UK; 6 INDEPTH Network, Accra, Ghana

**Keywords:** Heat, Cold, Temperature, Mortality, Socioeconomic factors, India

## Abstract

Ambient temperatures (heat and cold) are associated with mortality, but limited research is available about groups most vulnerable to these effects in rural populations. We estimated the effects of heat and cold on daily mortality among different sociodemographic groups in the Vadu HDSS area, western India. We studied all deaths in the Vadu HDSS area during 2004–2013. A conditional logistic regression model in a case-crossover design was used. Separate analyses were carried out for summer and winter season. Odds ratios (OR) and 95% confidence intervals (CI) were estimated for total mortality and population subgroups. Temperature above a threshold of 31 **°**C was associated with total mortality (OR 1.48, CI = 1.05–2.09) per 1 **°**C increase in daily mean temperature. Odds ratios were higher among females (OR 1.93; CI = 1.07–3.47), those with low education (OR 1.65; CI = 1.00–2.75), those owing larger agricultural land (OR 2.18; CI = 0.99–4.79), and farmers (OR 1.70; CI = 1.02–2.81). In winter, per 1 **°**C decrease in mean temperature, OR for total mortality was 1.06 (CI = 1.00–1.12) in lag 0–13 days. High risk of cold-related mortality was observed among people occupied in housework (OR = 1.09; CI = 1.00–1.19). Our study suggests that both heat and cold have an impact on mortality particularly heat, but also, to a smaller degree, cold have an impact. The effects may differ partly by sex, education, and occupation. These findings might have important policy implications in preventing heat and cold effects on particularly vulnerable groups of the rural populations in low and middle-income countries with hot semi-arid climate.

## Introduction

Global climate change is very likely increasing average temperature and impact of heat exposure in many places around the world (Costello et al. 2009; Kjellstrom et al. 2009; Stocker 2013). Scientific evidence of the relationship between weather/climate and health in low- and middle-income countries is well reported in sub-Saharan Africa and Asia including 12 INDEPTH Network surveillance sites (Smith 2014). Research in urban areas of low and middle-income countries has shown an increase in mortality in association with hot and cold weather (Hashizume et al. 2009; McMichael et al. 2008). Extreme heat events in 2015 were major health concerns in India and Pakistan, with more than 1000 attributed deaths during the summer when daily maximum temperature exceeded 40 °C (BBC 2015). The population of western India confronts frequent heat waves, with high risk of mortality and morbidity (Nag et al. 2013). However, there are relatively few studies of heat-related vulnerability specific to South Asian populations (Ingole et al. 2012; Tran et al. 2013). In particular, in rural India, heat and cold exposures have not been explored at large extent. Work-related heat stress has been studied in a handful of settings in India, e.g., outdoors, in poorly ventilated indoor workspaces, and near furnaces (Nag et al. 2013). Among slum dwellers in Gujarat, India, occupational exposure was independently related to heat illness (Tran et al. 2013). Commonly, work-related heat stress, and outdoor temperature load and its physiological impact associated with social and demographic parameters. Socioeconomic status is one important factor that may be a determinant of heat-related morbidity and mortality (Gronlund 2014). Recently the association between temperature and rainfall and mortality has been studied in the Vadu Health and Demographic Surveillance System (HDSS) area in India (Ingole et al. 2012, 2015). Individuals of low income are more likely to have chronic diseases or other medical risk factors, mental illness, and less adequate types of housing, which will all modify the risk of heat-related mortality. Therefore, research is needed to achieve better understanding of the multiple social and environmental factors of vulnerability to study interaction with heat exposures in the agricultural and manufacturing industry workers.

In this study, we aimed to quantify the association of heat during summer and cold during winter with mortality by sex, age, ownership of agricultural land, house type, education and occupation in Vadu Health and Demographic Surveillance System (HDSS), a rural setting in western India, during 2004–2013.

## Methods

### Study area

The Vadu HDSS is located in between latitude 18° 30 to 18° 47 N and longitude 73° 58 to 74° 12 E, covering a geographical area of 232 km^2^, with an average altitude of 560 m. Vadu HDSS includes 22 villages from two tehsils (administrative blocks) (14 villages from Shirur and 8 villages from Haveli) in Pune District of Maharashtra state. The total population was 131,545 (Vadu HDSS Database, August 2012) (Ingole et al. 2012, 2015). Vadu HDSS is a member of the International Network for the Demographic Evaluation of Populations and Their Health (INDEPTH) that conducts longitudinal surveillance for monitoring and evaluation of population health in low and middle-income countries (Sankoh and Byass 2012).

### Population data

The study data were obtained from the Vadu HDSS database for the period of January 2004 to December 2013. In Vadu HDSS, field research assistants (FRAs) visit each household and record demographic events twice a year (Ingole et al. 2015). Vadu HDSS collects information on deaths, births, in-out migration, and pregnancies during demographic surveillance rounds since 2003. In this analysis, we have used data of 3079 deaths; for each deceased, date of birth and date of death, gender, occupation, and education was available from routinely collected data. Additionally, the data on house type and land ownership were collected at household level during the socioeconomic status survey in 2004. These data were linked to each death record. Only individuals of age 15 years and older were included in the analysis.

### Classification of effect modifiers

Information on occupation is initially recorded as text in local language (Marathi) and later coded in the database. There were more than 30 different occupational classes, which we classified into five main groups based on local knowledge (farming, housework, manufacturing industry, service work, and others). Information on ownership of agriculture land (available for each household) was used. Owning 5 acres or more of agricultural land was considered high, while owning land smaller than 5 acres was medium, and owning no agriculture land was low.

Another effect modifier was the type of house, locally defined terms “pucca”, “semi-pucca” and “kachha”. Houses made from mud floor, thatched roof, walls painted with mud or cow dung are called kachha houses, houses that use material similar to kachha coupled with some material used for permanent structure such as walls with stones but still painted with mud or tin walls but definitely no concrete roof are called semi-pucca houses. Houses made of tiled floor, tin or concrete roof, and walls plastered with cement are called pucca houses. We classified all houses into either kachha (also including semi-pucca) or pucca.

Lastly, education was classified into three classes: no education or uncompleted primary school (low education), completed primary school (medium education), and completed secondary school or higher (including college and university) (high education).

### Weather data

Daily mean temperature for the study period of January 2004 to December 2013 was obtained from the National Oceanic and Atmospheric Administration (NOAA) (http://www.ncdc.noaa.gov). We also collected weather data from the local meteorological office of the Indian Meteorological Department (IMD) for the period of January 2004 to December 2012 to check its validity with online available data. We re-computed mean temperature in both datasets, which were reasonably highly correlated (*r* = 0.95). In this analysis, we have used daily mean temperature data that were collected from NOAA.

### Defining the heat and cold season

To find simple functions characterizing heat and cold effects in summer and winter respectively, we defined heat and cold season based on summer months and winter months. In the study area, the winter season lasts from November to February and is followed by summer that lasts from March to June. The monsoon in the study area starts from early June and continues until the beginning of October.

### Statistical methods

Analyses were carried out in two stages. In the exploratory stage, we used a quasi-Poisson regression model to examine the association of heat and cold with daily death counts over the study period of 2004–2013. We estimated the relationship of heat and cold with total mortality with a lag of 0–1 day in summer and 0–13 days in winter (Hashizume et al. 2008; McMichael et al. 2008). A cubic spline function was used to assess the functional form of the temperature-mortality relationship (six degrees of freedom (df) and to adjust for season and time trends. The exploratory analyses were done using the statistical software R-version 3.1.0.3 with splines fit by the package generalized additive model (GAM).

In the second stage, the dose-response relationship between temperature and mortality was explored for all deaths and for population subgroups based on the heat and cold function from the exploratory analysis using a time stratified case-crossover study design. Controls for each case were selected for the same year, month and day of the week as the case. Conditional logistic regression analysis was performed for each sub-group (effect modifiers), separately for heat (summer months) and cold (winter months) periods using statistical software STATA version 13. 1.

## Results

**Fig. 1:**
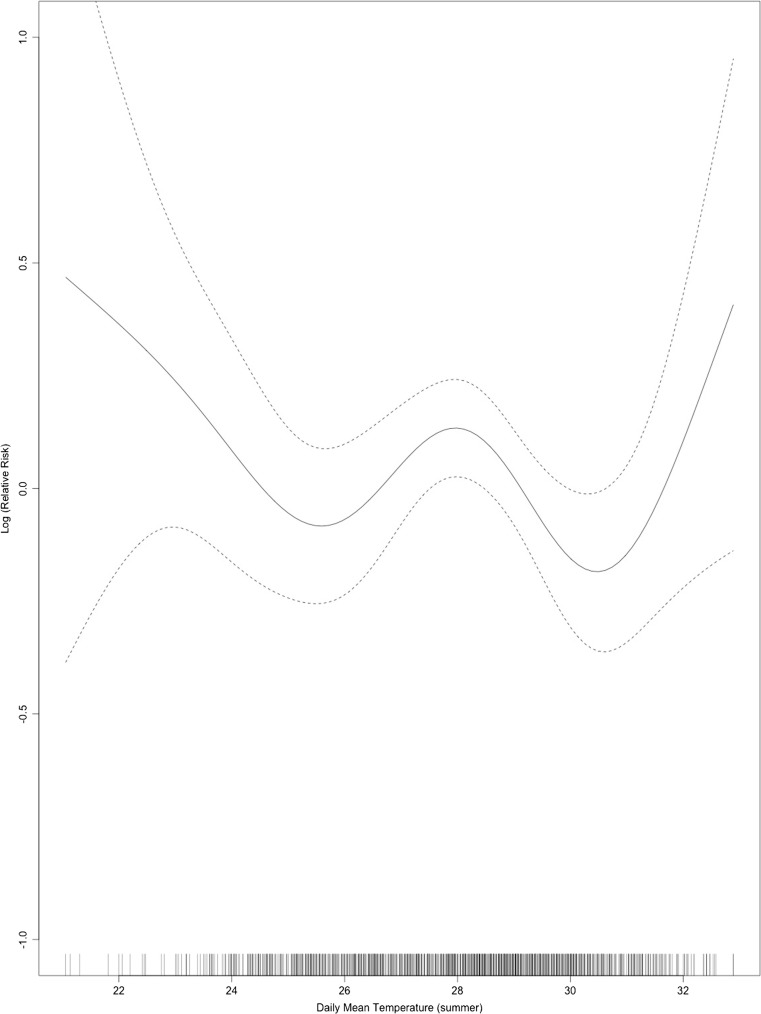
Association of daily mean temperature and total mortality (quasi-Poisson regression model) during summer period, lag 0–1 day

**Fig. 2:**
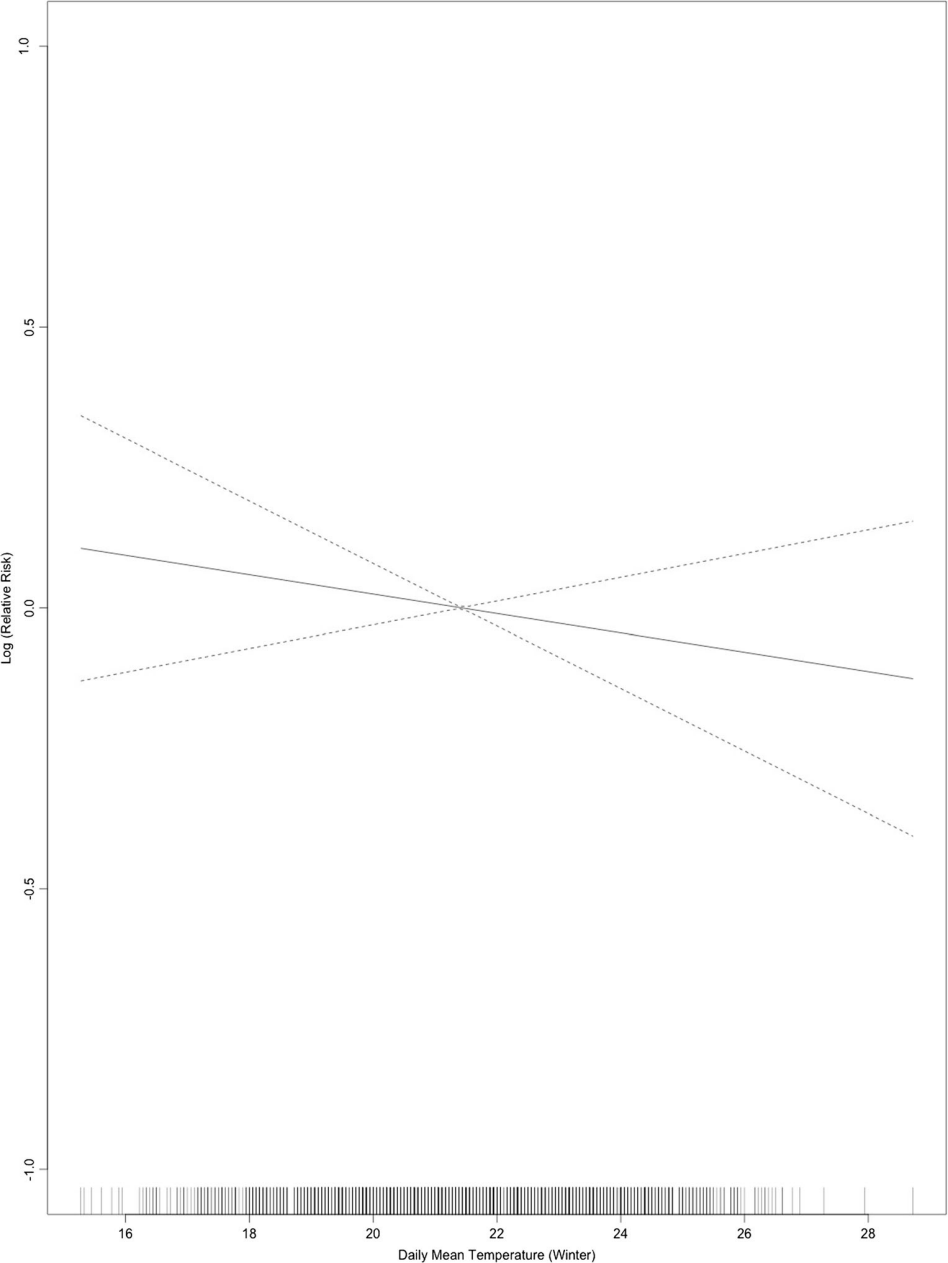
Associations of daily mean temperature and total mortality (quasi-Poisson regression model) during winter period, lag 0–13 days

Table 1 presents a summary of the daily mean temperature of summer and winter months. Table 2 displays descriptive statistics of all deaths and effect modifiers (occupation, education, land ownership, house type) used in this study. There were in total 3079 cases of deaths included in the analysis. In the summer period with lag 0–1, the temperature-mortality curve indicated a non-linear association with an upward slope above 31 **°**C (Fig. 1), for the winter season within lag 0–13 days, a linear association between daily mean temperature and total mortality was found (Fig. 2).

**Table 1:** Descriptive statistics of daily mean temperature (summer and winter months) during 2004–2013

Daily mean temperature (°C)	Mean	Std. Dev.	Min	Max
Summer months	27.9	2.2	21.0	33.2
Winter months	21.4	2.2	15.2	28.7

*°C* temperature in degree Celsius, *Std. Dev* standard deviation, *Min* minimum, *Max* maximum)

**Table 2:** Descriptive statistics of all deaths and population sub-groups during 2004–2013

Characteristics	No.	Percent
All deaths	3079	100
Age 15–67	1892	61.45
Age 68–80	732	23.77
Age 80+	455	14.78
Sex		
Male	1861	60.44
Female	1218	39.56
Occupation		
Farming	1131	36.74
House work	1388	45.09
Manufacturing work	230	7.47
Service work	222	7.21
Others	107	3.48
SES (agricultural land ownership)		
High (≥5 acres land)	730	32.6
Medium (<5 acres land)	1011	45.15
Low (no land)	498	22.24
House Type		
Kachha (poor quality)	901	40.24
Pucca (high quality)	1338	59.76
Education group		
Low (no or uncompleted primary school)	1360	44.17
Medium (completed primary school)	1094	35.53
High (completed secondary school)	625	20.3

In the second stage, we ran a conditional logistic regression model in a case-crossovers study design. For summer months, we found for total mortality an OR of 1.48 (CI = 1.05–2.09) per degree Celsius increase in daily mean temperature above the threshold of 31 °C in lag 0–1 day. Among occupational groups, the farmers showed the highest risk (OR 1.70, CI = 1.02–2.81). Residents with low education had a high risk as well (OR 1.66, CI = 1.00–2.73). Women had higher risk of heat-related mortality than men (OR 1.91, CI = 1.07–3.47). No differences between age groups were observed (Table 3).

**Table 3:** Risk of dying in summer above threshold 31 °C (lag 0–1 day), per degree Celsius increase, by age, sex, and other demographic parameters during 2004–2013 in Vadu HDSS, India

Characteristics	Odds ratio	95% Confidence interval
All Deaths	1.48	1.04–2.09
Age 15–67	1.51	0.97–2.35
Age 68–80	1.40	0.69–2.89
Age 80+	1.48	0.60–3.65
Sex		
Male	1.29	0.83–1.99
Female	1.93	1.07–3.48
Occupation		
Farming	1.70	1.03–2.81
House work	1.17	0.61–2.24
Manufacturing work	2.08	0.64–6.78
Other	1.61	0.45–5.71
Service work	0.89	0.18–4.39
Ownership of agricultural land		
High (≥5 acres land)	2.18	0.99–4.79
Medium (<5 acres land)	1.38	0.82–2.32
Low (no land)	1.25	0.44–3.58
House Type		
Kachha (poor quality)	1.87	0.99–3.54
Pucca (high quality)	1.35	0.82–2.25
Education Group		
Low (no or uncompleted primary school)	1.66	1.01–2.73
Medium (completed primary school)	1.75	0.94–3.26
High (completed secondary school)	0.86	0.36–2.02

Table 4 shows effect estimates during the winter season within lag 0–13 days. The total mortality and daily mean temperature in winter were found OR of 1.06 (CI = 1.00–1.12) per degree Celsius decrease in temperature up to 0–13 days. The effect of cold those employed in housework shows OR 1.09 (CI = 1.00–1.19). Elderly people (age 80+ years) had higher risk (OR 1.13, CI = 0.97–1.33) than younger ones (OR 1.05, CI = 0.98–1.13). No differences in heat and cold risks across sub-groups were statistically significant.

**Table 4:** Risk of dying in winter temperature (lag 0–13 days), per degree Celsius decrease, by age, sex and other demographic parameters during 2004–2013 in Vadu HDSS, India

Characteristics	Odds ratio	95% Confidence interval
All deaths	1.06	1.00–1.12
Age 15–67	1.05	0.98–1.13
Age 68–80	1.05	0.94–1.16
Age 80+	1.13	0.97–1.33
Sex		
Male	1.05	0.98–1.13
Female	1.05	0.97–1.16
Occupation		
Farming	1.06	0.97–1.16
House work	1.09	1.00–1.19
Manufacturing work	0.86	0.64–6.78
Other	1.06	0.87–1.27
Service work	1.10	0.84–1.45
Ownership of agricultural land		
High (≥5 acres land)	1.06	0.95–1.19
Medium (<5 acres land)	1.04	0.95–1.14
Low (no land)	1.02	0.89–1.16
House Type		
Kachha (poor quality)	1.04	0.94–1.15
Pucca (high quality)	1.05	0.89–1.16
Education Group		
Low (no or uncompleted primary school)	1.06	0.97–1.16
Medium (completed primary school)	1.04	0.96–1.14
High (completed secondary school)	1.08	0.96–1.22

## Discussion

In this study, we aimed to identify the social and environmental factors associated with heat and cold-related mortality in Vadu HDSS, western India, during 2004–2013. Confidence intervals for heat and cold risks for sub-groups were wide and overlapped, but there were differences that indicated suggestive evidence for some groups were more vulnerable than others. Specifically, people working in farming and individuals with no education or primary school uncompleted were at higher level of risk of dying on heat days. We found an increase in total mortality by 48% per degree Celsius above a threshold greater than 31 °C during the summer period. These findings are consistent with other research findings, which showed 43% increase in mortality, during the summer months in Gujarat in 2010 (Azhar et al. 2014; Ingole et al. 2015). In the present study, also, we found an increased risk for working age group (Ingole et al. 2012, 2015). Prior research in our population showed that men of working age were more vulnerable to heat than women, which suggests that occupational factors might contribute to heat-related mortality risk (Ingole et al. 2015). However, results show an increase of risk even for the wokring age group (15–67 years old), and especially among women.

Our results confirmed particularly high effects of heat on mortality among farmers (OR 1.70). This might be because in the present study, we investigated only heat during summer months, not all-year daily temperature means. Heat mortality risk varies by age and sex and shown that women at high risk. There may be physiological reasons for increased risk in women (Burse 1979; Havenith 2005; Kovats and Hajat 2008).

In Vadu HDSS population, individuals who had not completed primary school showed high risk of heat effects during the summer period. These associations may exist because people with little or no education may be less aware of health risk of heat or cold waves and high possibilities of work in farms. In the USA and China, scientists found that low-education level intensified the temperature-mortality relationship (Chan et al. 2012; Ma et al. 2012; O’Neill et al. 2003; Wang et al. 2014; Yang et al. 2012). Education, occupation, and land ownership of land may be indicator of socio-economic status. Social and demographic parameters, occupational heat exposure, and access to resources (e.g., water or health information) are likely to increase vulnerability (Tran et al. 2013). Therefore, future interventions (e.g., health education) targeted at specific population groups in rural India may reduce vulnerability to extreme heat (Ingole et al. 2015; Tran et al. 2013). Housing characteristics have also been related with heat-health outcomes (Maller and Strengers 2011). In our study, individuals living in kachha houses showed little vulnerability to heat during the summer period. In the Chicago heat waves in 1995 and 1999, housing characteristics were not found to be significant characteristics of vulnerability, after controlling for other characteristics of vulnerability (Gronlund 2014; Naughton et al. 2002; Semenza et al. 1996). The Vadu HDSS housing types might indicate indoor heat, but might also be associated with socioeconomic factors such as income or occupation.

In the cold season, overall results showed a 6% increase in total mortality per degree Celsius decreased temperature in lag 0–13 days. The cold effects on residents working in housework were higher than on those working as farmers and other occupational groups. This might be because of traditional cooking practices using biomass fuel that may keep the indoor environment warm (Fullerton et al. 2008; Wu et al. 2011). Elderly individuals had a higher cold-related mortality than younger ones in the cold season. Several studies have supported similar evidence that elderly individuals are most susceptible group (Basu 2009; Dang et al. 2016; Gasparrini et al. 2012; Hajat et al. 2007; Yang et al. 2012). This may be because aging induces a decrease in thermoregulatory abilities, and increased chronic diseases, which are likely to contribute to susceptibility to temperature in this group (Gasparrini et al. 2012). The cold effects on the population subgroups were not strongly associated. It might be that winter temperatures in Vadu usually are still comparably high and therefore pose little health threats (Ingole et al. 2015). More research is needed to improve understanding of the modulating factors such as housing quality, technology, local topography, urban design, and behavioral factors (Gronlund 2014; Ingole et al. 2015; Maller and Strengers 2011; McMichael et al. 2008). There were no clear modifications of cold effects in different population subgroups.

There are few strengths of this study that deserve consideration. This is the first study in a rural part of India, which focused on social and demographic characteristics and ambient temperature. Secondly, the HDSS platform offered a unique opportunity to exploit individual information that is rarely available in developing countries (Sankoh and Byass 2014). Some limitations of the study must be taken into account. There is no standard method to classify occupation groups for developing countries and other effect modifiers such as socio-economic status. There were too many occupation categories, some occupations could not be assigned to specific categories and these posed the problem of how to combine this diverse information into a five classes. Daily mortality and temperature data were available from 2004 to 2013, but information about socioeconomic status were obtained only at one point, in 2004. That means that allocation of socioeconomic factors might be outdated for those who died at a later point. Another challenge in this study was the rather small population size (very limited number of deaths in the population and in subgroups), which limits our statistical power and interpretation of effect estimates.

## Conclusion

This study suggests that effects of heat on total mortality in Vadu HDSS area are particularly high in women, residents working in Farms, and those with low education. This study underscores the need for increasing public awareness about effects of ambient air temperature on mortality. Local programs encouraging preventive measures, communicated through health education campaigns and community meetings, may mitigate such effects. Outdoor workers should be encouraged to avoid working during the hottest part of the day, wear appropriate clothing (lightweight) and bright colors, and to drink plenty of water. Although air-conditioning may also be protective, practical implementation would be limited to indoor environments and cost-prohibitive in many low resource settings. However, low-cost electric fans or cooling stations could be possible options for reducing heat stress. These findings should inform policy-makers of the vital importance of adaptation to and mitigation of ongoing climate change. Policy may rely on quantified evidence of the incremental impact of heat on health, particularly among vulnerable groups.
